# The student voice in quality assurance: what factors make for a great GP placement in the eyes of undergraduate medical students?

**DOI:** 10.1186/s12909-025-07500-4

**Published:** 2025-07-01

**Authors:** Simon Thornton, Trevor Thompson, Maria Gresham, Alice James

**Affiliations:** 1 Centre for Academic Primary Care, University of Bristol, Bristol, BS8 1UD UK; 2 Faculty of Health, University of Plymouth, Plymouth, UK

**Keywords:** Quality, Medical student, Feedback, General practice

## Abstract

**Background:**

With general practice becoming an increasingly important part of undergraduate medical education, it is vital to have reliable ways for assessing placement quality. The most prevalent tools for such assessment are the Dundee Ready Education Environment Measure (DREEM) and the Manchester Clinical Placement Index (MCPI). These instruments were not specifically designed for use only in GP contexts and were developed without student involvement at inception. As a result, they may omit quality indicators valued by students on GP placement. This study sought to understand the quality of learning in GP from the student perspective and compare this to what is assessed by DREEM and MCPI. We hope the results of this study will inform the development of placement quality questionnaires inspired by the student voice.

**Methods:**

Purposive sampling was used to recruit 17 undergraduate medical students in academic years 3–5 at the University of Bristol into four focus groups. These ran from May 2023 to April 2024 and were based on a topic guide developed and agreed on by all authors. Theoretical saturation was achieved as no new themes emerged in the final focus group. The focus groups were recorded and transcribed in full. The transcripts underwent systematic coding using NVivo. The codes formed the basis for the thematic analysis.

**Results:**

Four main themes emerged: a sense of belonging, quality of learning, efficiency of learning, and the qualities of the GP teacher. These themes are illustrated by pertinent quotes from the focus groups. Subthemes included being given appropriate clinical autonomy within the placement, timely access to resources, structured clinical activities, and tutor qualities such as enthusiasm and leadership.

**Conclusions:**

This paper offers a new perspective on the quality of GP placements by focusing on the student experience. It identifies themes and subthemes that education teams should assess, beyond what's covered by existing tools like MCPI and DREEM. These include students’ sense of belonging, perceived learning efficiency, and appropriate clinical autonomy. We argue that ignoring these areas neglects key aspects of student needs. We are piloting a new questionnaire incorporating these themes to better identify substandard placements and improve student experiences.

**Supplementary Information:**

The online version contains supplementary material available at 10.1186/s12909-025-07500-4.

## Background

Medical student teaching in general practice (GP) in 2020 was estimated to constitute 9.2% of medical curricula in the United Kingdom (UK) [1]. The proportion of teaching delivered in general practice is likely to increase significantly in the context of a proposed doubling of medical student numbers in the UK [2]. As the number of undergraduate teaching practices expands, agile quality assurance mechanisms will be important to help define and champion high standards of teaching, as we call on a community of practice who may be inexperienced as educators.

Different methods are used to assess the quality of undergraduate GP teaching environments [3]. This includes feedback (from students, teachers and patients), practice visits and using checklists to assess the suitability of a teaching practice against defined criteria. Cotton et al. proposed such a list of quality criteria for community-based education that covers four main domains: the physical environment, the learning environment, tutor characteristics and patient characteristics [4]. Similar criteria have been included in two prominent validated questionnaires for evaluating the educational environment in medical schools in both hospital and community settings: the Dundee Ready Education Environment Measure (DREEM) [5] and the shorter Manchester Clinical Placement Index (MCPI) [6]. Both have been used in community settings [6, 7] although only the MCPI was specifically developed for use in both community and hospital settings.

The DREEM questionnaire was designed by a Delphi panel of medical educators, rather than students, and whilst widely used, has been criticised for its length (50 items), lack of free text options and its validity in a clinical setting [8]. DREEM has been validated in international settings [9–13], although it has required adjustment in some cases, such as the removal of items [10, 13], which may limit its cross-cultural application. The development of the MCPI was grounded in educational theory and is a briefer 8 item questionnaire that includes free text responses and has been validated in both hospital and community settings [6] and internationally [9, 14]. Similar to DREEM, some limitations have been identified in the international context[9]. The MCPI appears to achieve equivalent discrimination between placements to the DREEM [15]. Hyde et al. compared student opinions of the MCPI and DREEM using free text questionnaire responses and a 7 point Likert scale and found that overall students appeared to favour the MCPI [8]. We were unable to find evidence that either of these questionnaires was developed with student involvement at inception.

During GP placement evaluations at the University of Bristol, we explored using DREEM and MCPI. We noticed themes arising from other feedback channels (such as emails and conversations) that were not captured by either of these. One example was the value students place on being offered appropriate levels of clinical *autonomy* on placement. All our students have supervised responsibility for patient care—but how this works out in practice varies from one setting to another. We wondered whether these themes were not addressed by DREEM or MCPI because these questionnaires are based on *faculty* perceptions of quality rather than notions of quality derived directly from student experience.

Some studies have explored student perspectives on GP placement quality [16]. Smith identified three main themes: GP tutor factors, practice factors and patient involvement [17]. When it comes to assessing the quality of student placements, it is easy to assume that the factors that make a quality practice are the same domains which students would want to cover in their feedback. There is evidence that students prefer providing feedback on aspects of their placement that can lead to tangible change [18]. For instance, a particular practice demographic may add much to student experience but, since this is not something within the power of any individual practice to change, it is less relevant to the students as a topic for feedback.

The aim of this study was to better understand placement quality by focusing on the features that current students consider most important and to compare these indicators to those assessed by DREEM and MCPI. Hopefully this study will inform the design of questionnaires that accurately reflect student priorities.

## Methods

We chose a focus group methodology to help students develop a shared conceptualization of what they value on placement. Any one individual may be constrained by their own particular experience, and the focus group allowed us to integrate perceptions across different placements and years of the course.

The University of Bristol medical programme is a five year undergraduate course, with the predominant clinical components occurring in years three to five. GP placements exist in all five years of the course. The GP placement in Year 3 consists of two, eight-day blocks, each in a different practice, as a group of six students. In Year 4, it is a longitudinal integrated clerkship (LIC) with 18 days in practice as group of four students. In Year 5, it is a nine-week block placement as a pair of students.

Purposive sampling was used to recruit students into four focus groups which were conducted from May 2023 to April 2024 with 17 University of Bristol medical students in academic years three to five. An email was sent to all students on placement, and those who responded were selected to take part. Year 3–5 students were chosen due to the amount of primary care that they had experienced by this stage in the course. Students were reimbursed for their time with an online shopping voucher. The composition of the focus groups is outlined in Table 1. Focus group sessions were of 60 min duration and organized around a topic guide agreed on by all authors. The participants initially undertook an ice-breaker exercise, ranking themes on cards which were derived from Cotton et al.’s quality criteria for community based medical education [4]. The students were then asked to reflect on their personal experiences of GP placements. The first three focus groups were facilitated by AJ and the fourth focus group was facilitated by ST and TT.

**Table 1:** Composition of the focus groups

Focus Group Number	Number of participants	Year of study
1	7	3
2	2	5
3	4	4 and 5
4	4	5

The focus groups were recorded and transcribed in full. The transcripts underwent systematic coding using NVivo. AJ, MG and ST analysed each of the transcripts independently. The codes formed the basis for the thematic analysis [19]. We believe we achieved theoretical saturation as no new themes emerged in the final focus group.

To protect the identity of participants, we have not documented which focus group they participated in, or which year of study they were in. The quotes are drawn from multiple students across all focus groups and years of study. Ethical approval was obtained from the University of Bristol Faculty of Health Sciences Research Ethics Committee.

## Results

**Fig. 1:**
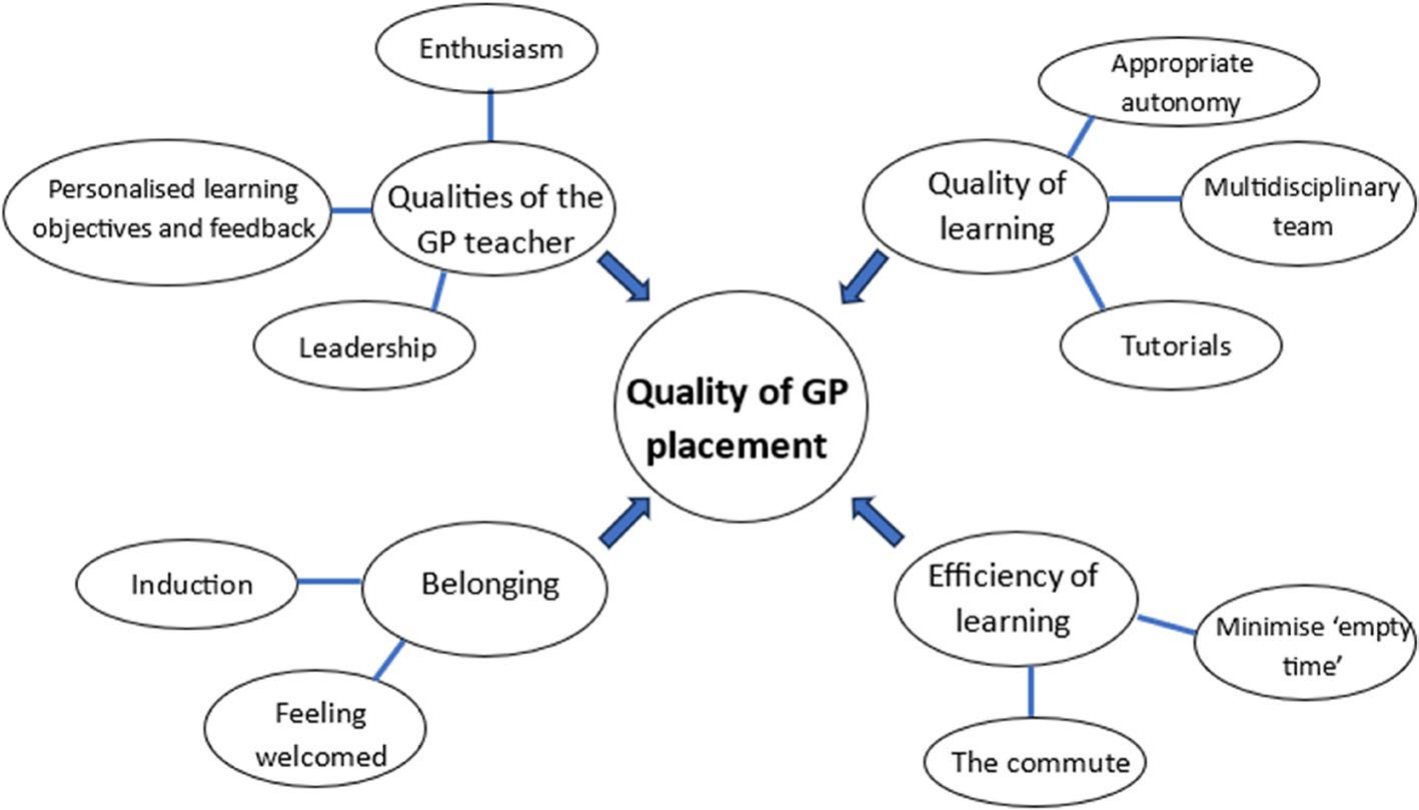
Thematic map

Four themes on quality emerged from the analysis and several subthemes. These are shown in Fig. 1. In the following sections, we explore each of these themes, drawing on selected quotations from the focus groups.

### Belonging

#### Induction

An induction was viewed as an essential part of developing a sense of belonging at the practice. As well as equipping students with key logistical information, and meeting staff, it allowed the opportunity for GP tutors to get to know the students personally and understand their individual learning needs.

*“…simply getting to know you at the start and talking about what your concerns are, I think that it can be so, so powerful. And that even just simply like stating that I want everyone to feel comfortable… I think it’s something that can be so simple and so powerful.”*

Students expressed frustration when they were not given timely access to computer systems, door codes and swipe cards.

*"[We have to] hover awkwardly when we're trying to go between rooms until someone can let us through… sometimes I feel like I'm getting in the way or I'm going to be late for something"*

#### Feeling welcomed

Students appreciated being incorporated into the routine of the practice. For example, breaks scheduled at the same time as other practice members allowed students to participate in informal social opportunities.

*“I quite liked having a schedule so that we knew what time you were having lunch and like what time our breaks were and I liked that those breaks were scheduled with other people.”*

Some students expressed frustration at practices where they didn’t feel welcome in communal spaces or where the practice didn’t have enough space for them.

*"[you’re] in the way… because you haven't been told that [they've] got space for you… so then we would leave and sometimes we sat in the car park… because there wasn't really any space for us"*

Students reported that a sense of belonging bolstered their agency to positively contribute to their practice:

*"… a practice where you're respected and you're treated as more of a colleague than perhaps, like a student, has been really beneficial. I think you're more motivated to work harder when you're treated in that way".*

### Quality of learning

#### Appropriate autonomy

Students appreciated the gradual shift towards independent practice that occurs as they become more experienced.

*“…what I quite appreciated this year was just having a bit more freedom to make decisions, and then check with the GP if that was what they would agree with.”*

Students found that consulting without their GP supervisor in the room (what one student referred to as ‘indirect clerking’) had its own merits:

*"I found the indirect clerking really useful in sort of practicing by myself just building a rapport with patients and getting my own style of consulting whereas I felt under a bit more pressure when I'm in direct consulting to make sure I'm ticking all the boxes… I did find the indirect was useful in those early Year 2 stages and just sort of getting comfortable with talking to patients and the whole feel of consulting."*

Students reflected that sometimes they felt like they were being given too much autonomy.

*“Often the GP wouldn't even come and see the patient, which I don't know if that's dodgy or not to be honest. If it felt dodgy I would say ‘can you come and see the patient?’ but I would say that it often felt like I was disturbing the GP who was meant to be supervising me.”*

It was clear that there was a ‘*Goldilocks zone’* of enough supervision that they felt well supported but had enough independence for them to develop their own consulting style and feel a degree of responsibility and autonomy.

*"… having the GP there but in a more supportive role… you can slowly sort of notice yourself over time doing more and more of the history and examination. And I think it definitely builds confidence and also having that safety net… if you do say something wrong"*

#### The multidisciplinary team

Students valued time spent with the multidisciplinary team but only when they were able to take a more active role.

*“Our most useful ones [sessions with allied health practitioners] have been ones where we can use it for clinical skills...when we're actively involved it’s really useful... I went with a paramedic through minor illnesses last year and that was quite useful because we never normally talk about like, thread worm or stuff”*

It was also important that members of the multidisciplinary team had been primed to expect the students.

“*Sometimes the other members of staff aren't really warned that we're going to be there…so they don't necessarily have it set up in a way that's going to be useful for us.”*

#### Tutorials

The delivery of core knowledge through tutorials in the GP setting was valued.

*“I found that I think based on the year and the year you're in and what was going on at the time, particularly for fourth and fifth year, I do think the content of the tutorials was often well targeted to what you needed to know.”*

Students also valued the personal experience that clinicians brought to their teaching.

*“The GP’s stories are quite useful as well…not a story but sort of their expertise…expanding on the different elements of it with their own experience is quite useful.”*

### Efficiency of learning

#### Minimise ‘empty time’

Students appreciated a clear structure to the day which maximizes the time spent in clinical activities and reduces the ‘empty time’ spent between clinical activities:

*"there is definitely at least half of my day that I think I haven't actually learned anything about anything valuable."*

The break between morning and afternoon surgeries in GP is often felt to be too long.

*“…previously, I've had lunch times that are two hours long, and it is frustrating because it feels like you could be doing something else with that time”*

Students valued the efficiency of learning in GP compared to hospital placements:

*“The GP learning was efficient… hospital learning is very fluffy… yes you have learnt, but it’s spread across like dust, a little bit here and there and you gather as you go.”*

The GP tutor played an important role in facilitating this efficiency:

*“…say you want to use that time in the middle of the day for studying and then she [the GP teacher] would very much just be like, how do you want me to support you in that? Do you want me to like, check in with you? Do you want me to quiz you on something? Or would you like to be kind of left alone and like, do your own little bit of studying and not hear from me, not a peep?”*

#### The commute from their accommodation

Travel to GP practices is a significant aspect of the placement that students want to be asked about. Students found that difficulties arose if they didn’t drive, didn’t have a car driver in their group or reliable transport links to practices.

*“You had to take 2 buses...I was always leaving the house two hours before the session even started...it just means that you show up a bit miserable and not really ready to learn.”*

Students felt it was important for practices to be flexible if there was a difficult commute.

*"If it's somewhere that's really hard to get to, then the structure of the day can be slightly different to allow people to get back early."*

### Qualities of the GP teacher

#### Enthusiasm

Students felt significantly influenced by the enthusiasm of their GP tutor. Enthusiasm served as an antidote to what could sometimes feel like a long and sedentary day:

*"I do struggle sitting down all day… so enthusiasm is a major thing… because then that will feed onto me."*

Frequently referenced was the GPs'supportive role in developing students'confidence to practice independently:

*"The more trust the GP gives us with clerking a patient or getting a history… I think the better. That's when I've got the most out of it."*

#### Leadership

Students also picked up on leadership skills demonstrated by their GP tutors.

*"[the GP] takes quite a lot of responsibility for the people in the practice… enjoying coming to work… he just emphasised the importance of creating a good work environment where people enjoy coming to work, and then you get the best out of people. And I think that's something that I really saw was being important and really felt part of"*.

#### Personalised learning objectives and feedback

Students valued a teacher who personalised teaching to them:

*“At the beginning of each session we would have like just 15 minutes to sit down, regroup, think about what we've been doing so far, what we feel less confident about and what we would like to get out of the day... after each patient, she would check in and be like, okay, how did you feel that went?... she really took the time to be like, each patient that you saw is about your learning and about you moving on.”*

Students felt it important to have the opportunity to feed back to the education team about how much direct observation of their consulting occurred on placement. Some students felt that the opportunity for direct observation by a senior clinician was almost unique to GP placements:

*"having someone to watch you [consult], it's the only time you really get someone to watch."*

As well as feedback on consultation skills, students were able to have their clinical findings corroborated by the GP:

*"it feels like on the wards you're sort of just taking a guess whereas then the GP can be like, oh, actually, I heard that too"*

## Discussion

With an increasing proportion of undergraduate teaching being delivered in GP, it’s important that any concerns over the quality of student placements are picked up and resolved early. We studied quality from a student perspective to ensure that we develop feedback methods that capture the most important aspects of GP placements through the student lens. High quality, comprehensive feedback allows us to identify and share areas of good practice, maintain high standards of teaching, and will help inspire the next generation of GPs.

Our students have identified four key themes from their undergraduate GP placements on which they valued the opportunity to provide feedback: a sense of belonging, the quality and efficiency of learning, and the attributes of the GP teacher. This is illustrated in the thematic map (Fig. 1). In this section we consider these themes, and their subthemes, in relation to the MCPI and DREEM questionnaires.

### Belonging

Previous work has recognized the importance of an induction or being made to feel welcome [4, 17], something that is assessed in both the MCPI and DREEM. From our focus groups, the concept of belonging was a strong theme that emerged. Whilst an induction and welcome can help foster a sense of belonging, they do not guarantee it. Belonging has been described as “*a subjective feeling of value and respect derived from a reciprocal relationship to an external referent that is built on a foundation of shared experiences, beliefs or personal characteristics”* [20]. It became apparent from our students that a sense of belonging was created not just by having an induction, or being made to feel welcome, but was a complex construct that evolved from interpersonal relationships with staff and patients throughout the practice, including their GP tutor. It has been argued that belongingness is an important component of participation that is easier to measure [21], and participation in a community of practice is a fundamental principle of situated learning [22]. Daniels et al. have developed a questionnaire specifically to assess belongingness amongst medical students, the Exeter Belongingness Assessment Tool (EBAT) [23] which can demonstrate differences in belongingness between different demographic groups [24]. For the purposes of quality assurance of placements, we feel that the EBAT is limited by its length (42 items) and its focus on belonging only.

### Qualities of the GP teacher

The importance of the qualities of the GP teacher are well recognized and our findings that students value an enthusiastic teacher are in keeping with other studies [4, 17]. Our students identified the importance of the GP teacher as a role model and leader, something that is felt to be an important process for the professional development of learners as well as shaping career choice [25]. Role modelling is recognized in other studies as an important characteristic of a good GP teacher [4, 26]. The importance of the GP as a leader is recognized in the MCPI [6] but not the DREEM questionnaire [5].

### Quality learning

Appropriate autonomy was an important facet of quality learning that emerged from our focus groups. This autonomy is achieved through an awareness of the learning needs of the student [17] and also through appropriate direction from the medical school. Autonomy is not something that is currently assessed by either MCPI or DREEM.

Park et al.’s meta-ethnography of GP teaching suggested that medical students are in pursuit of two kinds of information: knowledge to help them become good doctors, and facts required to pass their medical student exams [27]. Our students valued tutorials in GP as a useful way of acquiring these facts. Having tutorials in the GP practice is important to help dispel the view that hospital is the best setting for learning ‘textbook medicine’ for exams [27].

### Efficient learning

The fourth theme to emerge from our focus groups was efficiency of the learning time in practice. Multiple factors are at work in ensuring that time in practice is well spent, including the skills of the GP teacher, and any structure to the placement that is given by the medical school. Efficiency of learning included minimising any ‘empty time’ in the practice which commonly occurred in the gap between morning and afternoon surgery when GPs were busy with visits and administrative work. Students also expressed concerns about the length of the commute and the impact that this might have on their learning. The efficiency of learning is addressed by DREEM in the statement ‘*The teaching time is put to good use’* [5] and is addressed by MCPI under the statement *‘This placement was appropriately organised’* [6] which does not specifically assess the *efficiency* of learning and may be interpreted to mean a number of different things by students.

## Limitations

There were 17 focus group participants across four focus groups at one UK medical school. This study raises important themes that might be relevant to other institutions. Despite the small number of participants, many of the ideas emerging from the focus groups overlapped. To avoid moderator bias, a focus group topic guide was adhered to. As all four focus groups were conducted at the same medical school, it is likely that some of the participants knew each other and may have been less forthcoming with certain topics in front of their peers. 16 of the participants were female and one student was male which is not representative of the gender split of our student cohort. We did not record participants age, ethnicity or whether they were an international student. Future research involving a broader and more diverse sample would be beneficial to assess the extent to which these findings are generalizable.

## Conclusions

**Fig. 2:**
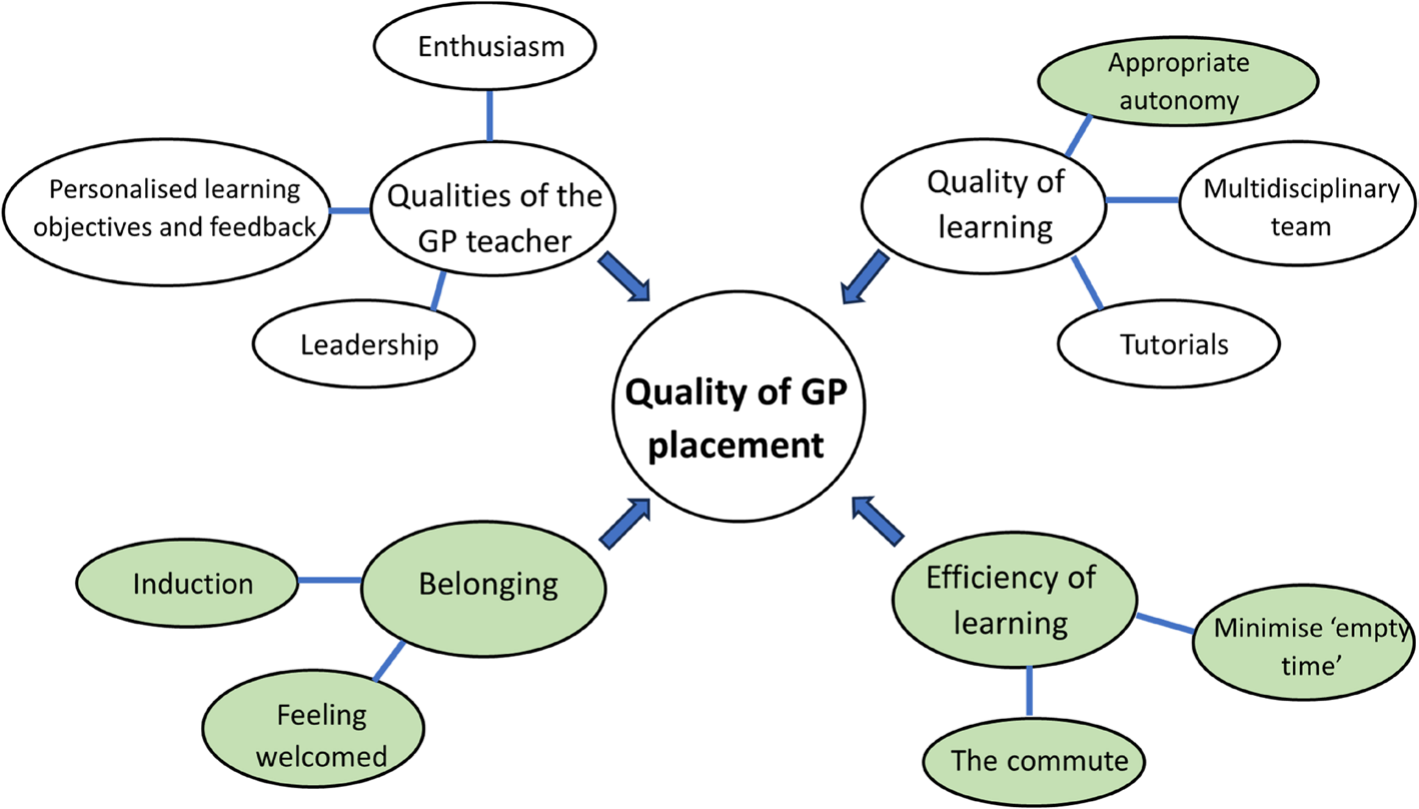
Thematic map. Topics not adequately assessed by current instruments highlighted in green

This paper contributes a fresh perspective on GP placement quality by listening directly to the student voice. We have distilled from our focus group data four themes that education teams involved in quality assurance should assess (Fig. 1). Though *qualities of the GP teacher* are covered in both MCPI [6] and DREEM [5] – the other three themes are not adequately assessed by current instruments. We recommend the development of tools that interrogate students’ sense of belonging on placement, their perceived efficiency of learning and the degree to which they are offered an appropriate level of clinical autonomy – an important subtheme of quality of learning. To exclude these areas (highlighted in green in the thematic map below, Fig. 2) is to fail to acknowledge areas of prime importance from the student perspective.

We are currently piloting a new questionnaire developed as a result of these focus groups which integrates findings from this study with other features seen in MPCI and DREEM.

We intend this new questionnaire to better enable us to identify settings which are not currently up to standard, and to make tailored interventions to improve the student experience. We would welcome the opportunity to collaborate with other teaching centers in the evaluation of this new questionnaire based on student perceptions of what helps them learn best in general practice.

## Supplementary Information

Supplementary Material 1

Supplementary Material 2

## Data Availability

The datasets generated during the current study are not publicly available as participants may be identifiable from the content of their discussion and have consented to publication of ‘quotes or parts of what I say’ but further data is available from the corresponding author on reasonable request.

## Abbreviations

DREEM: Dundee Ready Education Environment Measure
GP: General Practice
MCPI: Manchester Clinical Placement Index
UK: United Kingdom
