# Supplementary material for: The student voice in quality assurance: what factors make for a great GP placement in the eyes of undergraduate medical students?
Source: BMC Med Educ. 2025 Jul 1;25:943. doi: 10.1186/s12909-025-07500-4 (PMC12210570; doi:10.1186/s12909-025-07500-4)
Supplement: Supplementary file 1 — Supplementary Material 1 [file 12909_2025_7500_MOESM1_ESM.pdf]

University of BRISTOL
Centre for Academic Primary Care

GP Placement student feedback form

We would like to find out about your experience on GP placement. The following questionnaire was designed with the help of medical students. It should take around 10 minutes to complete. Your free-text comments are particularly important as they can lead directly to useful adjustments. Your feedback is not anonymous as we need to be able to contact you if you raise significant concerns. Your scores and comments will be reviewed by the central GP teaching team and may be passed on anonymously to your GP teacher. At the end of this form is a confidential box that you can use to record any concerns that you do not want to be passed on to your GP teacher.

GP Placement Details

1.What is the name of your practice?

2.Please enter your GP Practice ODS Code (please ask your GP teacher for this code). The code is a letter, followed by a series of numbers eg L38156.

3.Who has been your main GP teacher?

4.Please name any other regular GP teachers during your placement

5.Which clinical academy are you in?

Feedback for your GP teacher

6.Please enter any anonymised feedback that you would like us to pass on to your GP teacher. Your GP teachers really value your feedback, and are disappointed when they don't receive any

Your experience of your GP practice

7.I was made to feel **welcome** in the practice and I felt like I **belonged**. For example, being welcome in communal spaces, having an induction, having your point of view considered in meetings

Strongly disagree 1 2 3 4 5 Strongly agree

8.This GP placement **advanced my core medical knowledge** (including in preparation for assessment)

Strongly disagree 1 2 3 4 5 Strongly agree

9.My learning time in practice was **efficiently structured.** For example, time was efficiently used between surgeries.

Strongly disagree 1 2 3 4 5 Strongly agree

10.The level of **responsibility** that I was given was about right. By this we mean responsibility of managing your own clinical cases under supervision or independent patient contact.

Strongly disagree 1 2 3 4 5 Strongly agree

11.My placement felt **inclusive** for me and made reasonable adaptation for any additional needs that I have. For example, providing prayer space, accommodating any adjustments recommended in study support plans.

Strongly disagree 1 2 3 4 5 Strongly agree

12.The distance and **travel time** to the practice was acceptable.

Strongly disagree 1 2 3 4 5 Strongly agree

13.Did your placement include time spent with **allied health practitioners**? For example, pharmacists, physiotherapists, nurses. NB this is not part of the curriculum in Year 1 and 2

Yes No

14. The time spent with allied health professionals was a good use of my time on placement.

Strongly disagree 1 2 3 4 5 Strongly agree

15.I **enjoyed** my GP placement

Strongly disagree 1 2 3 4 5 Strongly agree

16.Any additional comments on your experience of your GP practice?

Your experience of your GP teacher

17.I received **high quality** teaching from my GP teachers

Strongly disagree 1 2 3 4 5 Strongly agree

18.My GP teachers were **enthusiastic**

Strongly disagree 1 2 3 4 5 Strongly agree

19.During the placement, I was provided with **high quality**, **individualised feedback**.

Strongly disagree 1 2 3 4 5 Strongly agree

20.My GP teacher shared an **authentic picture** of GP life. For example, discussing things that they enjoy about the career as well as things that they find more challenging.

Strongly disagree 1 2 3 4 5 Strongly agree

21.Any additional comments on your experience of your GP teacher?

The Central GP Teaching Team

22.**Communication** from the central university GP team was timely and useful. For example, information on where your placement was, any emails sent during the placement, One Note or Blackboard updates

Strongly disagree 1 2 3 4 5 Strongly agree

23.The **teaching materials** provided by the central teaching team were of high quality. This includes any course handbooks, email updates, Sway tutorials, One Note or Blackboard resources

Strongly disagree 1 2 3 4 5 Strongly agree

24.Any comments on the central GP teaching team?

CONFIDENTIAL SECTION

25.Please use this box to raise any concerns about your GP placement with the central GP teaching team in confidence. These comments will be reviewed by the GP year leads and will only be discussed with your GP teacher (if appropriate) after further discussion with you first.

