# Supplementary material for: The student voice in quality assurance: what factors make for a great GP placement in the eyes of undergraduate medical students?
Source: BMC Med Educ. 2025 Jul 1;25:943. doi: 10.1186/s12909-025-07500-4 (PMC12210570; doi:10.1186/s12909-025-07500-4)
Supplement: Supplementary file 2 — Supplementary Material 2 [file 12909_2025_7500_MOESM2_ESM.docx]

**Focus Group Topic Guide**

1. **Ice-breaker exercise**

At the start of the Focus Group, the facilitator will open by asking ‘what do you think are the most important things that we (University of Bristol) need to know about your GP placements to ensure that you are having the best possible experience?’

We will ask students to brainstorm in pairs or small groups for 2 minutes before feeding back to the facilitator with the aim of stimulating discussion. We will have the following themes on cards which the students can use as prompts if they wish:

- A thorough induction
- Teaching skills of the GP tutor
- Communication from the Primary Care teaching team
- Opportunity to complete assessments such as Case Based Discussions
- Opportunities to consult with patients

 However, blank cards will be provided for students to write down their own themes.

1. **Induction**

‘Tell me about your GP placement induction’

Ask students ‘did you receive an induction?’ if no discussion generated.

‘What makes a good GP placement induction?’

‘Tell us about any induction pitfalls you’ve come across’

1. **Facilities**

‘How important is the access to facilities as part of your GP placement experience? Facilities might include a rest room or study space, availability of computers or WiFi, kitchen facilities or storage space for belongings.’

1. **Placement activities**

‘What does a great GP tutorial look like?'

'Tell us about the style of supervision you’ve experienced whilst seeing patients’

1. **Experience**

‘What was or is excellent about your GP placement?’

‘What are the qualities of a GP Teacher that would make for a really good placement?’

‘What makes a bad day on GP Placement?’

‘What would you change about your GP placement?’

‘How important is it to feel like you belong? How best can a GP practice help you feel like you belong"?

‘How do GP placement days differ from hospital placement days?’

1. **Feedback**

‘How long should a feedback form be?’
